# Localized nuclear and perinuclear Ca^2+^ signals in intact mouse skeletal muscle fibers

**DOI:** 10.3389/fphys.2015.00263

**Published:** 2015-09-29

**Authors:** Tihomir Georgiev, Mikhail Svirin, Enrique Jaimovich, Rainer H. A. Fink

**Affiliations:** ^1^Medical Biophysics Unit, Institut für Physiologie und Pathophysiologie, Ruprecht Karls UniversitätHeidelberg, Germany; ^2^Facultad de Medicina, Center for Molecular Studies of the Cell, Universidad de ChileSantiago de Chile, Chile

**Keywords:** localized nuclear Ca^2+^ signals, localized perinuclear Ca^2+^ signals, global nuclear Ca^2+^ signals, hypertonic treatment, organelle Ca^2+^, satellite cells

## Abstract

Nuclear Ca^2+^ is important for the regulation of several nuclear processes such as gene expression. Localized Ca^2+^ signals (LCSs) in skeletal muscle fibers of mice have been mainly studied as Ca^2+^ release events from the sarcoplasmic reticulum. Their location with regard to cell nuclei has not been investigated. Our study is based on the hypothesis that LCSs associated with nuclei are present in skeletal muscle fibers of adult mice. Therefore, we carried out experiments addressing this question and we found novel Ca^2+^ signals associated with nuclei of skeletal muscle fibers (with possibly attached satellite cells). We measured localized nuclear and perinuclear Ca^2+^ signals (NLCSs and PLCSs) alongside cytosolic localized Ca^2+^ signals (CLCSs) during a hypertonic treatment. We also observed NLCSs under isotonic conditions. The NLCSs and PLCSs are Ca^2+^ signals in the range of micrometer [FWHM (full width at half maximum): 2.75 ± 0.27 μm (NLCSs) and 2.55 ± 0.17 μm (PLCSs), S.E.M.]. Additionally, global nuclear Ca^2+^ signals (NGCSs) were observed. To investigate which type of Ca^2+^ channels contribute to the Ca^2+^ signals associated with nuclei in skeletal muscle fibers, we performed measurements with the RyR blocker dantrolene, the DHPR blocker nifedipine or the IP_3_R blocker Xestospongin C. We observed Ca^2+^ signals associated with nuclei in the presence of each blocker. Nifedipine and dantrolene had an inhibitory effect on the fraction of fibers with PLCSs. The situation for the fraction of fibers with NLCSs is more complex indicating that RyR is less important for the generation of NLCSs compared to the generation of PLCSs. The fraction of fibers with NLCSs and PLCSs is not reduced in the presence of Xestospongin C. The localized perinuclear and intranuclear Ca^2+^ signals may be a powerful tool for the cell to regulate adaptive processes as gene expression. The intranuclear Ca^2+^ signals may be particularly interesting in this respect.

## Introduction

In skeletal muscle, Ca^2+^ signals lead to contraction through the dihydropyridine receptor (DHPR) and the ryanodine receptor (RyR), which are important ion channels for localized Ca^2+^ release in muscle fibers of mammals (Kirsch et al., 2001; Wang et al., 2005; Apostol et al., 2009; Pickering et al., 2009). They are located at the triad and the transverse tubules are a crucial part of the triad (Felder and Franzini-Armstrong, 2002). The transverse tubular system is a complex membranous network (Edwards and Launikonis, 2008; Jayasinghe and Launikonis, 2013; Jayasinghe et al., 2013) spanning all the volume of the muscle fiber and the large Ca^2+^ signals involved in the excitation-contraction coupling (ec coupling) process will often mask other processes mediated by Ca^2+^ in muscle fibers, which are not global and may be identified by a particular location.

Localized Ca^2+^ signals (LCSs) are present in many cell types (reviewed by Cheng and Lederer, 2008). In skeletal muscle fibers of adult mammals, LCSs mainly arising from the sarcoplasmic reticulum occur in a significant number after chemical or mechanical skinning (Kirsch et al., 2001; Zhou et al., 2003) or after an osmotic treatment in intact skeletal muscle fibers (Wang et al., 2005; Teichmann et al., 2008; Pickering et al., 2009; Tjondrokoesoemo et al., 2013). Such LCSs were also found after an osmotic treatment in intact skeletal muscle fibers from mdx mice (Wang et al., 2005; Teichmann et al., 2008). Mdx mice are an animal model for human Duchenne muscular dystrophy (Bulfield et al., 1984).

Nuclear Ca^2+^ regulates important processes such as gene expression (Hardingham et al., 1997). The transcriptional repressor downstream regulatory element antagonist modulator (DREAM) is regulated by nuclear Ca^2+^ (Carrión et al., 1999). Nuclear Ca^2+^ induces phosphorylation of the transcription factor cAMP response element binding protein (CREB) in isolated nuclei of myotubes (Cárdenas et al., 2005). There is also structural evidence for perinuclear Ca^2+^ microdomains in cardiac myocytes (Escobar et al., 2011) and for an independent nuclear Ca^2+^ release machinery in different cell types (Malhas et al., 2011; Resende et al., 2013). Furthermore, the Ca^2+^ release channels ryanodine receptor (RyR) and inositol 1,4,5-triphosphate receptor (IP_3_R) are present in intranuclear regions of C2C12 cells and myotubes, respectively (Cárdenas et al., 2005; Marius et al., 2006). Additionally, nuclear Ca^2+^ is important for tumor growth (Rodrigues et al., 2007).

Other important LCSs at the nucleus (localized nuclear Ca^2+^ signals: NLCSs) and localized perinuclear Ca^2+^ signals (PLCSs) were measured in cardiac myocytes and Purkinje cells, respectively (García et al., 2004; Yang and Steele, 2005; Hirose et al., 2008; Luo et al., 2008). LCSs were also observed in close proximity to the nucleus of HeLa cells followed by a transient nuclear Ca^2+^ increase (Lipp et al., 1997). Global nuclear Ca^2+^ signals (NGCSs) were recorded in different cell types including isolated nuclei of myotubes (Jaimovich et al., 2000; Echevarría et al., 2003; Cárdenas et al., 2005; Zima et al., 2007; Elsing et al., 2012; Ibarra et al., 2013) and it has recently been demonstrated that in cardiac muscle cells there are fast nuclear Ca^2+^ signals occurring independently to the cytosolic Ca^2+^ signals (Ibarra et al., 2013). Recently, it has been found that the IP_3_R1 (inositol 1,4,5-triphosphate receptor type 1) is required for the induction of LCSs in skeletal muscle fibers of mice after a hypotonic treatment (Tjondrokoesoemo et al., 2013). Single channel recordings of the IP_3_R in isolated nuclei of muscle cells have been described (Kusnier et al., 2006) and a large conductance, IP_3_ sensitive channel in the inner membrane of nuclei has recently been reported in adult skeletal muscle (Yarotskyy and Dirksen, 2014).

Localized nuclear and perinuclear Ca^2+^ signals have not been described in skeletal muscle fibers. In particular, localized intranuclear Ca^2+^ signals are very interesting as they can target specific subnuclear regions and regulate subnuclear processes (Echevarría et al., 2003). Those signals may be important in the regulation of adaptive processes. Therefore, we searched for localized intranuclear Ca^2+^ signals in mouse skeletal muscle fibers.

In this study, we investigated LCSs in skeletal muscle fibers of adult mice during a hypertonic treatment and simultaneously recorded the signals of a DNA dye or a membrane dye for identification of their nuclear origin.

## Material and methods

### Sample preparation, solutions, and blockers

Wild type C57 (wt) mice, aged 10–48 weeks, were sacrificed according to §1 and §4 Abs. 3 Tierschutzgesetz from 29.05.1998 (approved by the Regierungspräsidium Karlsruhe). Skeletal muscle fibers with possibly attached satellite cells from the interossei muscles of the toe were enzymatically isolated using collagenase following a protocol similar to that described in an earlier study (Friedrich et al., 1999). The isolation was performed in the following isotonic solution (used by Wang et al., 2005): NaCl 140 mmol/l, KCl 5 mmol/l, CaCl_2_ 2.5 mmol/l, MgCl_2_ 2 mmol/l, HEPES 10 mmol/l, pH 7.2, ~290 mOsm. The skeletal muscle fibers were stored in this isotonic solution and used on the same day.

For the measurements of LCSs, the skeletal muscle fibers were stained with the Ca^2+^ indicator Fluo-4 AM (Molecular Probes, Eugene, OR, USA; 10 μmol/l) and the DNA dye HCS NuclearMask Deep Red stain (Molecular Probes, volume ratio 1/1000–1/3000) for 15–30 min at 37°C or with Fluo-4 AM (10 μmol/l) and the membrane dye di-8-ANEPPS (Sigma Aldrich, St. Louis, MO, USA; 40 μmol/l) for 15–20 min at 37°C.

As LCSs are rarely observed under isotonic conditions (Kirsch et al., 2001; Teichmann et al., 2008), we used a hypertonic solution as a stimulus. The highest frequency of LCSs in skeletal muscle fibers can be achieved under hypertonic treatment with a solution containing 50 mmol/l CaCl_2_ (Teichmann et al., 2008). In the present study we investigated LCSs associated with the nucleus. The nuclear surface is small compared to the cytosolic surface and a high frequency of LCSs simplifies the investigation of LCSs associated with the nucleus. For those reasons we used the following hypertonic solution: NaCl 140 mmol/l, KCl 5 mmol/l, CaCl_2_ 50 mmol/l, MgCl_2_ 2 mmol/l, HEPES 10 mmol/l, pH 7.2, ~420 mOsm (used also by Wang et al., 2005).

In some measurements the following hypertonic solution containing sorbitol was used as a stimulus: NaCl 140 mmol/l, KCl 5 mmol/l, CaCl_2_ 2.5 mmol/l, MgCl_2_ 2 mmol/l, HEPES 10 mmol/l, sorbitol 150 mmol/l, pH 7.2, ~440 mOsm.

Most measurements were performed with a hypertonic solution containing 50 mmol/l CaCl_2_ and this solution will be described as the hypertonic solution. It will be specially noted when the hypertonic solution containing sorbitol was used.

For the measurements with nifedipine (Sigma Aldrich; 100 μmol/l as used by Pickering et al., 2009), the skeletal muscle fibers were preincubated with nifedipine for 10–30 min and then the hypertonic solution also containing nifedipine was added.

In other experiments the skeletal muscle fibers were preincubated with dantrolene (Sigma Aldrich; 100 μmol/l) for 30–60 min before the hypertonic dantrolene solution was added.

For measurements with Xestospongin C (Sigma Aldrich; 10 μmol/l), the skeletal muscle fibers were preincubated with Xestospongin C for 30–40 min and the hypertonic solution containing Xestospongin C was added.

For the identification of the satellite cells, the skeletal muscle fiber preparations were exposed to anti-CD34-FITC (RAM34, rat IgG, BD Pharmingen, San Jose, CA, USA) in a volume ratio 1/200 for 3 h at 37°C as described by Liu and Schneider (2014). During the last 30 min of the 3 h the Ca^2+^ indicator Rhod-2 AM (2 μmol/l, Molecular Probes) and the DNA dye HCS NuclearMask Deep Red stain (volume ratio 1/3000) were added.

### Optical setup

An inverted microscope (DM IRBE, Leica Microsystems, Mannheim, Germany), a confocal laser scanning unit (SP2, Leica), a 63X water immersion objective (PL APO 63X/1.20W CORR, Leica), the laser line 488 nm of an Argon laser and the laser lines 543 and 633 nm of a HeNe laser were used. The fluorescence was detected on a descanned light path by a photomultiplier tube (R9624, Hamamatsu, Hamamatsu City, Japan).

XYT images were saved as 8-bit images and contain 512 ∗ 512 pixels with pixel sizes between 0.186 and 0.232 μm. Each image was recorded within 0.82 s.

XT 8-bit images were recorded with a time resolution of 800 lines per second or 1000 lines per second. The pixel size is 0.063 μm and the images contain 512 pixels in x-direction.

The images shown in the figures were converted from 8 bit to RGB.

### Measurement procedure

The dye-loaded skeletal muscle fibers were placed on Poly-L-lysin (Sigma Aldrich) coated coverslips. The different combinations of dyes, the different laser lines used for each combination, the detected spectral ranges and the maximal deviations from the stated detected ranges are summarized in Table 1 (see Supplementary Material for more details).

**Table 1:** **The table summarizes the combinations of dyes, the laser lines, the detection channels, and the maximal deviations from the stated detected ranges used for the investigation of Ca^2+^ signals associated with nuclei**.

**Dye combination**	**Laser lines (nm)**	**Detection channels (nm)**	**Maximal deviation from the stated detection ranges (nm)**
Fluo-4 AM and HCS NuclearMask Deep Red stain	488 and 633	500–600 and 650–800	3
Fluo-4 AM and HCS NuclearMask Deep Red stain	488	500–600 and 650–800	0
Fluo-4 AM and di-8-ANEPPS	488	500–550 and 650–800	4
Anti-CD34-FITC and Rhod-2 AM and HCS NuclearMask Deep Red stain	488 and 633	500–530 and 700–800	0
Anti-CD34-FITC and Rhod-2 AM and HCS NuclearMask Deep Red stain	543 and 633	550–620 and 700–800 or 555–615 and 700–800	0

In a few experiments with Fluo-4 AM and HCS NuclearMask Deep Red stain only the laser line 488 nm was used for the excitation of both dyes. We performed these experiments to exclude the possibility of chromatic errors of the optical system because of the different wavelengths used for excitation. Even in conditions when the DNA dye was excited first with 633 nm and then with 488 nm we could not observe a dislocation of the nucleus. In all figures presented here the DNA dye was excited with 633 nm.

Microspheres (see next chapter) were excited with 488 nm and the spectral ranges 500–600 and 650–800 nm were simultaneously detected and no shifts could be observed. These observations show that if the same laser line was used for excitation no chromatic shifts are detectable. Small lateral shifts (less than the lateral resolution) could be observed in some experiments when the microspheres were excited with 488 nm detecting the spectral range 500–600 nm and then excited with 633 nm detecting the spectral range 650–800 nm. Such shifts could not be observed as described above when the outlines of nuclei were compared using 488 nm (detected spectral range 650–800 nm) and 633 nm (detected spectral range 650–800 nm) for excitation.

For most measurements, after the addition of the hypertonic solution or the hypertonic solution containing sorbitol first a structural measurement of the DNA dye signal or the di-8-ANEPPS signal was recorded taking averages of eight images. Then the image sequence of the structural signal and the Ca^2+^ signal were measured simultaneously. After this measurement the same structural measurement as at the beginning was recorded and this measurement is again an average of eight images. This image was compared with the first image and the structural images recorded simultaneously with the Ca^2+^ signal to determine if there was a displacement of the fiber during the measurement. In some cases, the signals of the DNA dye or the membrane dye recorded simultaneously with the Ca^2+^ measurements were too noisy. If not specifically mentioned the DNA dye signal or the di-8-ANEPPS signal shown in the figures are the signals recorded before or after the measurement of the Ca^2+^ signal.

In some measurements an average of eight images of the Ca^2+^ signal was recorded simultaneously with the structural measurements at the beginning and at the end. These records were performed for structural purposes since some nuclei could be observed in the Fluo-4 AM signal due to elevated fluorescence in the nuclear envelope.

For one of the four recorded NGCSs, the signal of the DNA dye was solely recorded before and after the measurement, and not simultaneously.

All measurements were performed not later than 70 min after the addition of the hypertonic solution.

### Axial and lateral resolution of the optical system and Z stacks of the nucleus

We used microspheres with a diameter of 0.1 μm (TetraSpeck™ Microspheres, 0.1 μm, Fluorescent Blue/Green/Orange/Dark Red, Molecular Probes) diluted in water to determine the lateral and axial resolution of the optical system.

For the determination of the lateral and axial resolution z stacks of the microspheres were performed and the Fiji plugin MetroloJ (Matthews and Cordeliéres, 2010) was used.

After the measurements of the LCSs, z stack images of the nuclei were taken as due to the limited axial resolution of the optical setup, it is possible that the measured LCSs within the nucleus in the two dimensional records could have been originated outside of the nucleus. To demonstrate that the LCS is indeed inside the nucleus, a signal of the DNA dye should be recorded inside the region of the LCS in a plane above and under the plane of the Ca^2+^ measurement with a minimal distance *d*_m_ assuming *d*_m_ is a sufficient distance criterion. The axial resolution of the optical system was determined with measurements of microspheres as described before. For the measurement of the Ca^2+^ signal the following settings were used: excitation 488 nm, detection 500–600 nm and the axial and lateral resolutions for those settings are: *r*_axial;488;500−600_ = (2.19± 0.12) μm and *r*_lateral;488;500−600_ = (0.48± 0.03) μm (for a pinhole diameter of 70 μm, 3.15 Airy units) and *r*_axial;488;500−600_ = (1.67± 0.16) μm and *r*_lateral;488;500−600_ = (0.44± 0.02) μm (for a pinhole diameter of 50 μm, 2.24 Airy units). For the record of the DNA dye signal the following settings were used: excitation 633 nm, detection 650–800 nm and the axial and lateral resolutions for those settings are: *r*_axial;633;650−800_ = (1.78± 0.08) μm and *r*_lateral;633;650−800_ = (0.48± 0.02) μm, (for a pinhole diameter of 70 μm, 3.15 Airy units) and *r*_axial;633;650−800_ = (1.62± 0.12) μm and *r*_lateral;633;650−800_ = (0.44± 0.05) μm (for a pinhole diameter of 50 μm, 2.24 Airy units). The minimal distance *d*_m_ for two dyes with two different axial resolution limits is: *d*_m_ = 1∕2(*r*_axial;488;500−600_ + Δ*r*_axial;488;500−600_ + *r*_axial;633;650−800_ + Δ*r*_axial;633;650−800_). For our settings *d*_m_ is 2.09 μm for a pinhole diameter of 70 μm (3.15 Airy units) and 1.79 μm for a pinhole diameter of 50 μm (2.24 Airy units).

It cannot be excluded that partly clusters of more than one microsphere were measured and this could lead to a somewhat lower calculated axial resolution (larger r_axial_ and *d*_m_) compared to the true axial resolution of the system (Cole et al., 2011).

### Image analysis and analysis of LCSs

For image analysis we used ImageJ, Fiji, and WCIF ImageJ (Schindelin et al., 2012; Schneider et al., 2012).

The LCSs were identified with the ImageJ plugin xySpark (Steele and Steele, 2014). The spark threshold coefficient was set to 3.6, the Gaussian fit filter threshold to 0.5 and the spatial filter threshold to 10.

LCSs are referred as NLCSs if their centroid is located inside the nucleus in the two dimensional image (when identified by the experimenter and the algorithm). The NLCSs and CLCSs, used to determine the properties of the NLCSs and CLCSs (determined by the algorithm) and the nuclear and cytosolic frequencies of LCSs, were identified by the algorithm and visually confirmed by the experimenter. Signals identified solely by the algorithm or visually by the experimenter are not included.

CLCSs and intranuclear Ca^2+^ signals (INCSs: NLCSs and NGCSs) used to determine the fraction of responding fibers and nuclei were only identified by the experimenter as the occurrence of “false negatives” was more critical for this analysis and several NLCSs were restricted by the boundaries of the nucleus providing further challenge for the detection with the algorithm. When LCSs were solely identified by the experimenter, they are referred as NLCSs if they are located inside the nucleus in the two dimensional image. Additionally, the fraction of NLCSs is stated in Table 2 (in brackets) when only NLCSs simultaneously identified by the algorithm and by the experimenter were considered.

**Table 2:** **(A) Fraction of cells and nuclei responding with CLCSs, PLCSs, NLCSs, NGCSs, and INCSs in skeletal muscle fibers. For the fraction of cells and nuclei responding with NLCSs and INCSs two different values are stated. For the determination of the first value NLCSs identified by the experimenter were considered. For the determination of the second value (in brackets), NLCSs identified by the experimenter and by the algorithm were considered. (B) Number of investigated images per fiber and nucleus for the determination of the fraction of fibers responding with CLCSs, NLCSs, NGCSs, INCSs or PLCSs and NLCSs (when considering NLCSs identified by the algorithm and by the experimenter). Means ± S.E.M. (C) Averaged number of investigated nuclei, fibers and mice for the determination of the fraction of fibers responding with CLCSs, NLCSs, NGCSs, INCSs or PLCSs and NLCSs (when considering NLCSs identified by the algorithm and by the experimenter)**.

		**CLCSs (%)**	**PLCSs (%)**	**NLCSs (%)**	**NGCSs (%)**	**INCSs (%)**
**(A)**						
**Hypertonic**	Cells	66	31	16 (6)	13	29 (19)
	Nuclei	–	21	5 (2)	3	8 (5)
**Hypertonic**	Cells	62	18	23 (9)	0	23 (9)
**dantrolene**	Nuclei	–	6	7 (3)	0	7 (3)
**Hypertonic**	Cells	56	0	11 (0)	6	17 (6)
**nifedipine**	Nuclei	–	0	3 (0)	2	5 (2)
**Hypertonic**	Cells	73	45	18 (18)	0	18 (18)
**XestosponginC**	Nuclei	–	17	4 (4)	0	4 (4)
**Isotonic**	Cells	13	0	6 (6)	0	6 (6)
	Nuclei	–	0	2 (2)	0	2 (2)
			**CLCS, NLCSs, NGCSs, and INCSs**		**PLCSs and NLCSs**	
**(B)**						
**Hypertonic**	Averaged number of images per fiber		293 ± 27		248 ± 28	
	Averaged number of images per nucleus		285 ± 13		269 ± 13	
**Hypertonic dantrolene**	Averaged number of images per fiber		359 ± 23		295 ± 21	
	Averaged number of images per nucleus		319 ± 18		280 ± 10	
**Hypertonic nifedipine**	Averaged number of images per fiber		340 ± 20		291 ± 34	
	Averaged number of images per nucleus		307 ± 12		257 ± 19	
**Hypertonic Xestospongin C**	Averaged number of images per fiber		382 ± 31		382 ± 31	
	Averaged number of images per nucleus		409 ± 19		409 ± 19	
**Isotonic**	Averaged number of images per fiber		43 ± 2		43 ± 2	
	Averaged number of images per nucleus		44 ± 1		44 ± 1	
**(C)**						
**Hypertonic**	Number of nuclei		127		121	
	Number of fibers		32		32	
	Number of mice		13		13	
**Hypertonic dantrolene**	Number of nuclei		43		35	
	Number of fibers		13		11	
	Number of mice		4		4	
**Hypertonic nifedipine**	Number of nuclei		60		36	
	Number of fibers		18		13	
	Number of mice		3		3	
**Hypertonic Xestospongin C**	Number of nuclei		47		47	
	Number of fibers		11		11	
	Number of mice		2		2	
**Isotonic**	Number of nuclei		46		46	
	Number of fibers		16		16	
	Number of mice		13		13	

NLCSs marked in the graphs were identified by the algorithm and visually by the experimenter.

LCSs are referred as PLCSs if the distance between their centroid and the nucleus is ≤ 2 μm (for records in xyt images). PLCSs were identified by the algorithm and also visually by the experimenter. The dataset used for the analysis of PLCSs is somewhat smaller than the dataset used for the analysis of INCSs since datasets with small displacement during the measurement (for instance during the addition of the hypertonic solution) could not be analyzed with the algorithm. In xt images PLCSs were identified by the experimenter.

## Results

### NLCSs and NGCSs in intact mouse skeletal muscle fibers

**Figure 1:**
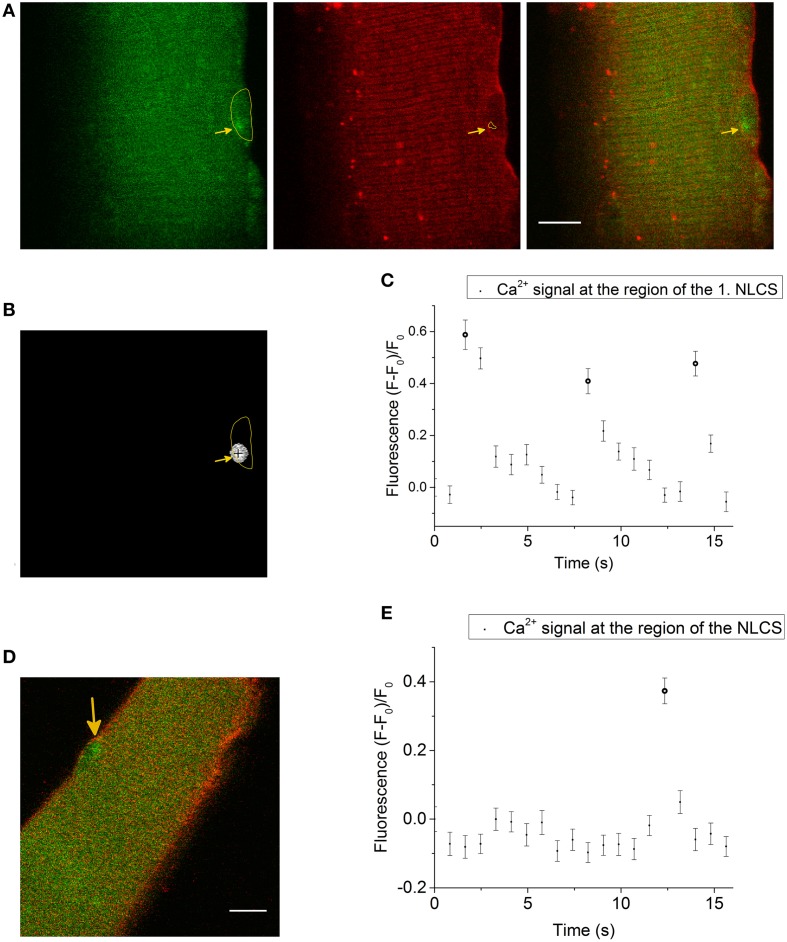
**(A)** LCS in a skeletal muscle fiber stained with the Ca^2+^ indicator Fluo-4 AM (left side, green) and the membrane dye di-8-ANEPPS (second image, red). The last image is a merge of both signals. The yellow marked region in the first image is a nuclear shaped structure in the measurement of the di-8-ANEPPS signal. A LCS is located inside the nuclear shaped compartment (arrow in the first image). The arrow in the second image shows at the region of the NLCS (yellow marked region). The measurement was recorded after the addition of the hypertonic solution. Scale bar 10 μm. **(B)** The cross marks the centroid coordinates of the LCS shown in **(A)** and the yellow marked region is the nuclear shaped compartment from **(A)**. **(C)** Fluorescence of the Ca^2+^ signal at the region of the first NLCS [presented in **(A)** and **(B)**]. Three NLCSs could be observed with a similar location and the fluorescence intensities of the images where a NLCS appears are marked as NLCS. **(D)** Skeletal muscle fiber stained with Fluo-4 AM (green) and di-8-ANEPPS (red). A LCS in a nuclear shaped compartment is shown (arrow). The Ca^2+^ signal and the di-8-ANEPPS signal were recorded simultaneously. The LCS was measured under isotonic conditions. Scale bar 10 μm. **(E)** Fluorescence of Fluo-4 AM at the region of the NLCS presented in **(D)**. The NLCS is marked.

We simultaneously recorded the signal of the Ca^2+^ indicator Fluo-4 AM and the membrane dye di-8-ANEPPS and found LCSs in nuclear shaped compartments of the fiber (Figures 1A,B,D). The nuclear region of skeletal muscle fibers is mostly free from the tubular system (Supplementary Material Image 1). Absence of the tubular system in the nucleus of skeletal muscle fibers has been shown by Jayasinghe and Launikonis (2013).

The NLCS in Figure 1A was recorded under hypertonic treatment. The NLCS presented in Figure 1D was measured under isotonic conditions and in a skeletal muscle fiber from a different animal. The NLCSs presented in Figure 1C can be observed on consecutive images. The NLCS presented in Figure 1E could be recorded only in one image.

**Figure 2:**
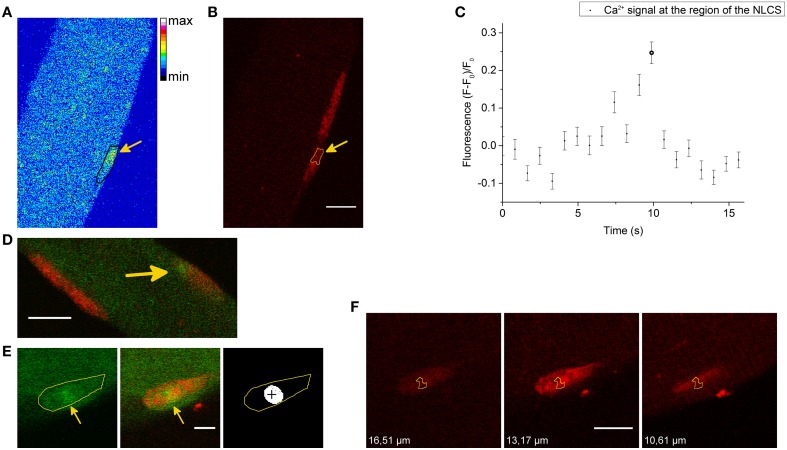
**(A)** NLCS in a skeletal muscle fiber stained with Fluo-4 AM and the DNA indicator HCS NuclearMask Deep Red stain. The black marked region is the nucleus and the arrow indicates a NLCS. The measurement was recorded after the addition of the hypertonic solution. **(B)** Signal of the DNA dye in the same part of the skeletal muscle fiber presented in **(A)**. The arrow indicates the position of the NLCS (yellow marked region). Scale bar 10 μm. **(C)** The graph shows the Ca^2+^ signal at the region of the NLCS presented in **(A)**. The fluorescence of the NLCS presented in **(A)** is marked as NLCS. **(D)** NLCS (arrow) in a skeletal muscle fiber stained with Fluo-4 AM (green) and the DNA dye (red). The NLCS was recorded under hypertonic treatment. Scale bar 10 μm. **(E)** NLCS in a skeletal muscle fiber stained with Fluo-4 AM (green) and the DNA dye (red). The first image (left side) is the Ca^2+^ measurement showing a NLCS. The region of the nucleus is the yellow marked region. The second image is a merge of the DNA signal and the Ca^2+^ signal. The arrows show at the NLCS. The cross (**right image**) marks the centroid of the NLCS. The record was obtained after the addition of the hypertonic solution. Scale bar 5 μm. **(F)** Three images of the nucleus in three different z planes are presented. The yellow marked region is the region of the NLCS shown in **(E)**. The z position is written on each image. The NLCS was recorded in the z plane of the second image (13.17 μm). Scale bar 10 μm.

We also recorded NLCSs under hypertonic treatment using the DNA dye HCS NuclearMask Deep Red stain to more specifically identify their nuclear origin. Figures 2A,B,D,E show NLCSs in three different fibers from different animals. Before the NLCS presented in Figure 2A was recorded a complex signaling pattern could be observed (Figure 2C). Alongside the NLCSs we observed many CLCSs.

The NLCS presented in Figure 2E is approximately in the center of the nucleus. The cross in the last image of Figure 2E marks the centroid calculated by the algorithm and it is located inside the nucleus. Using z-stacks of the nucleus, we could demonstrate that at least part of the NLCS is indeed inside the nucleus (Figure 2F). In Figure 2F, three different z planes of the nucleus are shown. The z plane of the NLCS recorded during the hypertonic treatment (Figure 2E) is the second image and the yellow marked surface is the region of the NLCS. The first (left side) and the last (right side) images are z planes above and under the z plane of the NLCS. The difference between those z planes and the z plane of the NLCS is greater than *d*_m_ = 2.09 μm for a pinhole diameter of 70 μm, which was used for this measurement. Part of the NLCS is located inside the nucleus in those planes. A similar observation was obtained from a second NLCS located inside another nucleus of that fiber. Furthermore, several NLCSs were restricted by the boundaries of the nucleus in two dimensional images. Therefore, it can be concluded that at least part of the recorded NLCSs is located definitely inside the nucleus.

**Figure 3:**
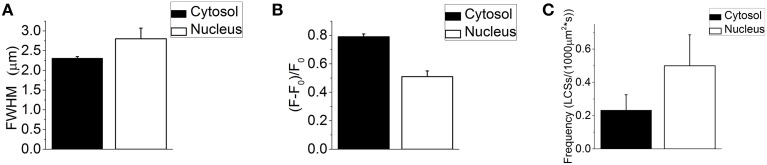
**(A)** Mean FWHM of 878 LCSs in the cytosol (seven fibers with NLCSs, seven mice) and 36 NLCS (nine nuclei, seven fibers, seven mice) recorded during the hypertonic treatment. **(B)** Mean amplitude (*F* – *F*_0_)/*F*_0_ of 878 LCSs in the cytosol (seven fibers with NLCSs, seven mice) and 36 NLCS (nine nuclei, seven fibers, seven mice) recorded during the hypertonic treatment. **(C)** Cytosolic and nuclear frequency of LCSs recorded in seven skeletal muscle fibers with NLCS during the hypertonic treatment (seven mice). The stated values are Means ± S.E.M.

Thirty-seven NLCSs in eight fibers (seven animals) out of 41 fibers (16 animals) were identified by the algorithm and visually by the experimenter. Ten NLCSs in two fibers were identified when using di-8-ANEPPS. Twenty-seven NLCSs in six fibers were identified when using the DNA dye to visualize the nuclei. One NLCS was measured under isotonic conditions and 36 NLCSs after the addition of the hypertonic solution. The mean full width at half maximum (FWHM) is (2.75 ± 0.27) μm and the mean amplitude (*F* – *F*_0_)/*F*_0_ is 0.55 ± 0.05. When the NLCS measured under isotonic conditions is excluded from the statistics, the properties of the 36 NLCSs recorded after the addition of the hypertonic solution are: the mean FWHM is (2.80 ± 0.27) μm and the mean amplitude (*F* – *F*_0_)/*F*_0_ is 0.51 ± 0.04. The properties of the 878 LCSs (seven fibers with NLCSs) detected in the cytosol of fibers with NLCSs during the hypertonic treatment are the mean FWHM: (2.31 ± 0.04) μm and the mean amplitude (*F* – *F*_0_)/*F*_0_: (0.79 ± 0.02). Those results are summarized in Figures 3A,B.

The frequencies (*F*) of LCSs in the nucleoplasm and in the cytosol of fibers and nuclei with NLCSs are: *F*_Cytosol_: (0.233 ± 0.093) LCSs/(1000 μm^2*^ s) and *F*_Nucleus_: (0.500 ± 0.186) LCSs/(1000 μm^2*^ s) (Figure 3C).

NLCSs were visually identified in three further fibers (in one skeletal muscle fiber using di-8-ANEPPS and in two skeletal muscle fibers using the DNA dye for the visualization of the nuclei) by the experimenter but not by the algorithm.

**Figure 4:**
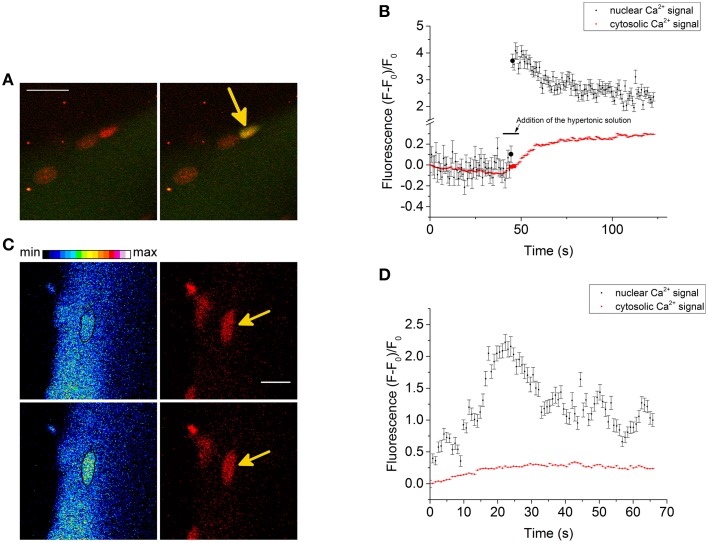
**(A)** NGCS in a skeletal muscle fiber stained with the Ca^2+^ indicator Fluo-4 AM (green) and the DNA dye HCS NuclearMask Deep Red stain (red). The two images are a merge of the signals of the Ca^2+^ and DNA indicator. Both signals were recorded simultaneously. The images were recorded during the addition of the hypertonic solution and the time difference between the images is 0.82 s. Scale bar 20 μm. **(B)** The graphs show the nuclear and cytosolic fluorescence of the Ca^2+^ signal. During the addition of the hypertonic solution, there is a shrinkage of the skeletal muscle fiber. We choose a small region of interest that was inside the nucleus and a second region of interest that is inside the fiber (but not inside the nucleus) during the whole measurement. The larger circles in the graph are the nuclear and cytosolic fluorescence intensities of the Ca^2+^ signal of the images shown in **(A)**. **(C)** NGCS in another skeletal muscle fiber (different animal) stained with the Ca^2+^ indicator Fluo-4 AM (green) and the DNA dye (red). The images on the left side are images of the Ca^2+^ signal and their time difference is 1.64 s. The black marked regions are the coordinates of one nucleus. The images on the right side are the corresponding measurements of the DNA signal. The images were recorded after the addition of the hypertonic solution and the signals of the Ca^2+^ and the DNA indicator were detected simultaneously. Scale bar 10 μm. **(D)** The graphs show the nuclear and cytosolic fluorescence intensity of the Ca^2+^ signal against time. The second image in **(C)** shows the Ca^2+^ signal briefly after the increase of the Ca^2+^ signal (second point in the graph). Because of the hypertonic condition there is a shrinkage of the cell. To measure the nuclear and cytosolic fluorescence of the Ca^2+^ signal, we used two small regions of interest. One of them was located within the nucleus and the other one within the cytosol.

We also recorded NGCSs in four skeletal muscle fibers out of 41 fibers during the hypertonic treatment. In Figure 4A a NGCS during the addition of the hypertonic solution is shown. In Figure 4B, a graph showing the nuclear and cytosolic fluorescence of the Ca^2+^ signal is presented. We observed NGCSs in nuclei with a similar location (at the periphery of the muscle fiber) in two further fibers.

Figure 4C shows another NGCS in a skeletal muscle fiber of a different animal after the addition of the hypertonic solution. Interestingly, one can observe an oscillating behavior of the nuclear Ca^2+^ signal (Figure 4D).

In two cells located in close proximity to a fiber (but not attached), likely to be satellite cells, we observed global cellular Ca^2+^ signals.

We determined the fraction of fibers and nuclei responding with CLCSs (only for the fibers), INCSs, NLCSs and NGCSs under isotonic conditions and during the hypertonic treatment (Table 2). Not included are the results of nine fibers since during those measurements only fibers with an activity of CLCSs were selected. NLCSs were identified in five out of those nine fibers by the algorithm and by the experimenter.

Usually NLCSs were observed in nuclei at the periphery of skeletal muscle fibers. In one skeletal muscle fiber one NLCS (identified by the algorithm and visually by the experimenter) and in a further skeletal muscle fiber from a different animal NLCSs (identified visually by the experimenter) were observed in central nuclei. Using z-stacks we showed that those are central nuclei.

We identified INCSs in 15 fibers. In four fibers with NGCSs, we observed in each fiber only in one nucleus NGCSs and no other Ca^2+^ signals associated with nuclei were observed. (4 ± 1) nuclei per fiber were investigated. In nine out of 11 fibers with NLCSs we observed NLCSs only in one nucleus in each fiber. In two fibers, we observed NLCSs in two nuclei of each fiber. (5 ± 1) nuclei per fiber were investigated.

### PLCSs in intact mouse skeletal muscle fibers

**Figure 5:**
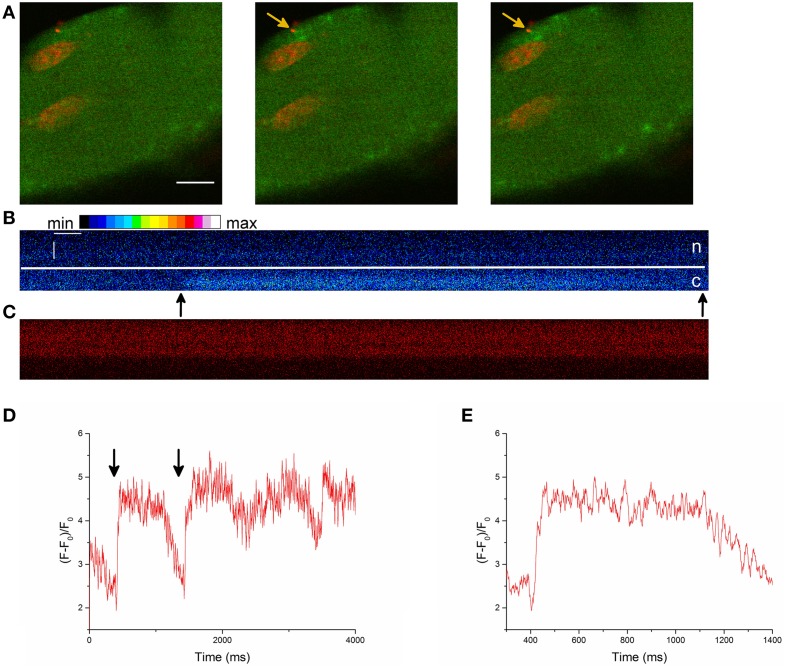
**(A)** Part of a skeletal muscle fiber stained with the Ca^2+^ indicator Fluo-4 AM and the DNA dye HCS NuclearMask Deep Red stain. The images are a merge of the Ca^2+^ signal (green) and the DNA dye signal (red). The time difference between the images is 0.82 s. An activity of PLCSs can be observed. It appears that the nucleus acts as a border for the localized Ca^2+^ signals. The record was obtained after the addition of the hypertonic solution. Scale bar 10 μm. **(B)** Line scan image of a PLCS in a skeletal muscle fiber stained with Fluo-4 AM and the DNA indicator. The Fluo-4 signal is presented. The arrows point at the beginning and at the end of the PLCS. Scale bars: 50 ms (horizontal) and 2 μm (vertical), c is the cytoplasm, n is the nucleus and the line marks the border of the nucleus. **(C)** The simultaneously recorded signal of the DNA indicator from the skeletal muscle fiber shown in **(B)**. For presentation purposes only intensities of the pixels between 0 and 110 can be differentiated, a few pixels with intensities higher than 110 have the same brightness in the presentation. **(D)** Fluorescence at the position of the PLCS (arrows at the beginning and end of the PLCS) presented in **(B)**. The graph shows a larger part of the measurement presented in **(B)**. Several PLCSs can be observed. For presentation purposes 10 pixels were averaged. **(E)** Magnified section of **(D)** at the location of the first PLCS.

Under hypertonic treatment, we observed PLCSs in 10 skeletal muscle fibers (from four animals) out of 32 fibers (from 13 animals; ~ 31%). Twenty-five nuclei out of 121 nuclei were associated with PLCSs (~21%; Table 2). Figure 5A shows a PLCS in a skeletal muscle fiber stained with Fluo-4 AM and the DNA indicator HCS NuclearMask Deep Red stain.

Our observations suggest that PLCSs occur more frequently than NLCSs in skeletal muscle fibers (Table 2).

The properties of the 58 detected PLCSs are: FWHM (2.55 ± 0.17) μm and (*F* – *F*_0_)/*F*_0_ = (0.99 ± 0.06).

To get some insight concerning the duration and spatiotemporal properties of the PLCS we recorded xt images with a higher temporal resolution (Figures 5B–E). We recorded PLCSs from two nuclei from different fibers and found a FDHM (full duration at half maximum) of (1250 ± 720) ms (Mean ± S.E.M. for three PLCSs).

### The RyR blocker dantrolene and the DHPR blocker nifedipine have each an inhibitory effect on LCSs associated with the nucleus

To investigate the possible involvement of the RyR and the DHPR in the genesis of LCSs associated with the nucleus, we performed experiments in the presence of the RyR blocker dantrolene or the DHPR blocker nifedipine during a hypertonic treatment.

The results are summarized in Table 2. Those observations show that the occurrence of PLCSs is reduced in the presence of each blocker showing that the DHPR and RyR are important for the generation of PLCSs in skeletal muscle fibers during a hypertonic treatment.

In the presence of each blocker, we observed NLCSs during the hypertonic treatment (Table 2). It is likely that the recorded NGCSs in the presence of nifedipine are located in a nucleus of a satellite cell, as in the measurement of the Ca^2+^ signal a border of a weaker Ca^2+^ signal could be observed between the nucleus and the muscle fiber. The fraction of fibers and nuclei with NLCSs is not decreased in the measurements with dantrolene. However, the observed NLCSs in the presence of each blocker have small amplitudes, except a NLCS in one fiber in the presence of dantrolene. This is also reflected in the fact that in the presence of dantrolene one NLCS could be simultaneously identified by the experimenter and by the algorithm (Table 2, values in brackets). In the presence of nifedipine, none of the NLCSs identified in two fibers by the experimenter could be identified by the algorithm (Table 2, values in brackets).

These observations suggest that PLCSs are more strongly inhibited in the presence of each blocker compared to INCSs.

**Figure 6:**
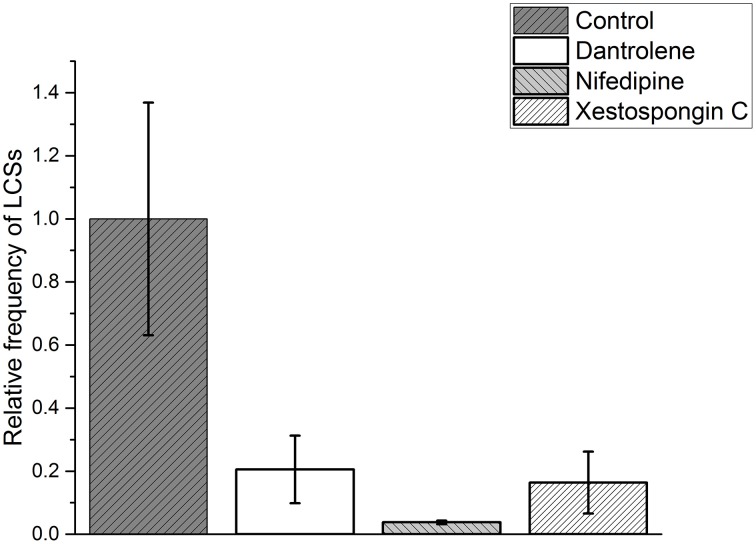
**Relative frequency of cellular localized Ca^2+^ signals (cellular LCSs) in skeletal muscle fibers of mice after the addition of a hypertonic solution in the absence of a blocker (control, 33 cells from 13 mice) and in the presence of dantrolene (11 cells from four mice) or nifedipine (13 cells from three mice) or Xestospongin C (11 cell from two mice)**. The stated values are Means ± S.E.M.

We normalized the frequency (*F*_Control_) of cellular LCSs in skeletal muscle fibers after the addition of the hypertonic solution to 1.000 ± 0.369. In the presence of dantrolene the relative frequency of LCSs was reduced to 0.205 ± 0.107. The effect of nifedipine was even stronger and reduced the relative frequency of LCSs to 0.038 ± 0.006. The results are summarized in Figure 6 (see Supplementary Material for the criteria used to determine the cellular frequencies of LCSs).

### The IP_3_R blocker xestospongin C does not reduce the fraction of fibers with NLCSs and PLCSs

We performed experiments in the presence of Xestospongin C to investigate a possible role of the IP_3_R in the generation of LCSs associated with nuclei of skeletal muscle fibers and of cellular LCSs. The fraction of fibers with NLCSs is not decreased in the presence of Xestospongin C and the fraction of nuclei with NLCSs (when identified by the experimenter) is slightly decreased (Table 2). Five NLCSs were identified in two nuclei of two different fibers (from two mice). Four of those NLCSs were confirmed by the algorithm. In one of the nuclei we observed four NLCSs (three NLCSs identified by the algorithm and the experimenter). This stronger activity is similar to the activity of some nuclei under control conditions. Nuclei with such activity could not be observed in the presence of nifedipine or dantrolene. The fraction of fibers with PLCSs is not decreased in the presence of Xestospongin C (Table 2). The fraction of nuclei with PLCSs is slightly decreased (Table 2). On the other hand, the frequency of cellular LCSs is decreased in the presence of Xestospongin C (Figure 6).

### Satellite cells and NLCSs in skeletal muscle fibers

It cannot be excluded that the nuclear Ca^2+^ signals occur (partly) in nuclei of satellite cells attached to skeletal muscle fibers.

**Figure 7:**
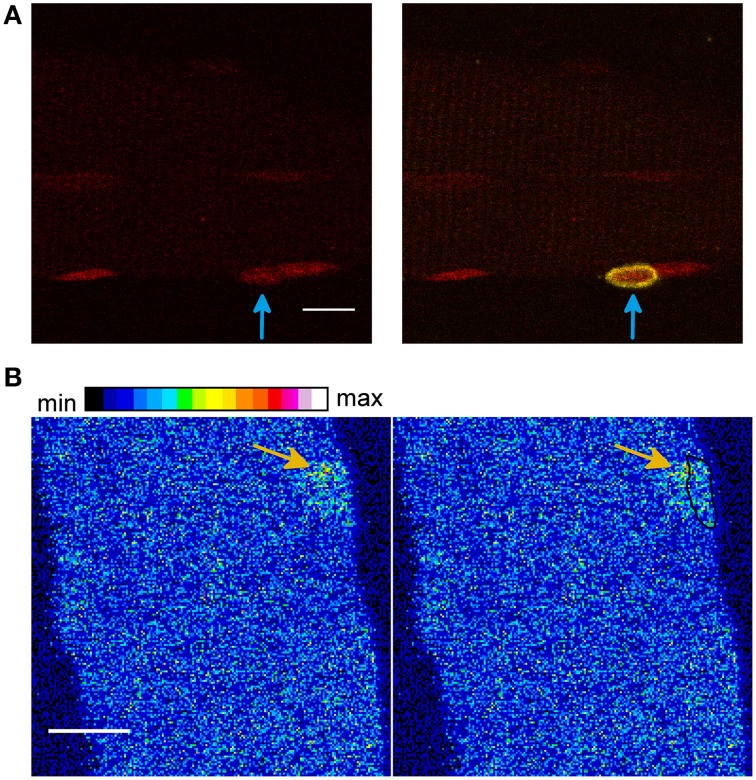
**(A)** A satellite cell (yellow) attached to a skeletal muscle fiber. The measurement was obtained after the addition of the hypertonic solution. The skeletal muscle fiber was stained with a marker for satellite cells (yellow, signal shown in the merged image, right side), the DNA dye HCS NuclearMask Deep Red stain (red, signal shown on the **left image** and in the merged image, right side) and the Ca^2+^ indicator Rhod-2 AM (signal not shown). It can be observed that one of the nuclei belongs to a satellite cell (arrow in the **right image**, merge of both signals). Scale bar 10 μm. **(B)** Skeletal muscle fiber stained with the Ca^2+^ indicator Fluo-4 AM and the DNA dye HCS NuclearMask Deep Red stain. The first image (right side) shows the Ca^2+^ signal and the second image shows the Ca^2+^ signal and the coordinates of the nucleus (black marked region). A LCS in close proximity to the nucleus can be observed (arrow). The measurement was obtained after the addition of the hypertonic sorbitol containing solution. Scale bar 10 μm.

Therefore we used a marker for the satellite cells, as recently described by Liu and Schneider (Liu and Schneider, 2014). Figure 7A shows a skeletal muscle fiber stained with a marker for satellite cells (yellow), the DNA dye (red), and the Ca^2+^ indicator Rhod-2 AM (signal not shown). The measurement shows that one of the nuclei belongs to a satellite cell.

Using Rhod-2 AM, NLCSs were observed in two skeletal muscle fibers from two animals after the addition of the hypertonic solution. These NLCSs were identified by the algorithm and by the experimenter. We could not detect a signal of the satellite cell marker surrounding those nuclei. Sixty-four nuclei without the satellite cell marker of 14 fibers from five animals and five nuclei of satellite cells attached to four fibers from three animals were investigated under the hypertonic treatment.

We observed one NLCS identified by the algorithm and by the experimenter in a nucleus not surrounded by the satellite cell marker under isotonic conditions. Twenty nuclei without the satellite cell marker of five fibers from four animals and one nucleus of a satellite cell attached to one fiber were investigated under isotonic conditions.

### LCSs in skeletal muscle fibers during a hypertonic treatment with a sorbitol containing solution

Since the Ca^2+^ concentration of the hypertonic solution used as a stimulus is increased compared to the isotonic solution, we performed experiments with a sorbitol containing hypertonic solution providing a more physiological Ca^2+^ gradient between the cytosol and the extracellular solution.

We observed CLCSs in nine fibers (from four animals) out of 23 fibers (from four animals).

In one skeletal muscle fiber, we observed two LCSs located in close proximity to the nucleus. One of those signals is shown in Figure 7B. Small part of the LCS is located inside the nuclear surface. In another fiber of a different animal, we observed one LCS in close proximity (< 2 μm) to the nucleus. Those signals were solely identified by the experimenter. Near nuclear LCSs were observed in ~9% of the fibers (two out of 23) and ~2% of the nuclei (2 out of 110).

After the addition of the hypertonic solution containing sorbitol we observed a global Ca^2+^ signal in three fibers (three animals) out of four fibers (four animals) and this signal may mask the LCSs during the first minutes. Similar observations have been described in skeletal muscle fibers after the addition of sucrose or mannitol containing hypertonic solutions (Pickering et al., 2009).

## Discussion

We describe here to our knowledge for the first time a variety of Ca^2+^ signals associated with the nucleus of mammalian adult muscle fibers. In particular, the NLCSs may be of interest. They are subnuclear Ca^2+^ signals and may represent an important tool for the regulation of subnuclear processes, as it was suggested by Echevarría et al. (2003) and more recently by Resende et al. (2013). They may be important for the regulation of adaptive processes as gene expression. Additionally, PLCSs may be involved in the regulation of gene transcription as suggested in a previous study for Purkinje cells (Hirose et al., 2008). In another study, it has been demonstrated that local perinuclear Ca^2+^ signaling is important for excitation-transcription coupling in cardiac myocytes and those Ca^2+^ signals were not detectable with Fluo-3 or Rhod-2 (Wu et al., 2006). In the context of Ca^2+^ signals which may be too small to be detected with standard fluorescent Ca^2+^ indicators, we speculate here that the NLCSs and PLCSs presented in our study may also occur spontaneously (under isotonic conditions) in a significant number but they may become more abundant (and their properties may change) during the stressing stimulus so they become easier to detect. Interestingly, we observed also NLCSs in skeletal muscle fibers under isotonic conditions. But we could not observe PLCSs or NGCSs under isotonic conditions. Additionally, we observed LCSs in two fibers in close proximity to the nucleus under hypertonic treatment with a solution containing sorbitol, leading to a less stressful condition and keeping the physiological Ca^2+^ gradient between the cytosol and the extracellular solution. The observations obtained under isotonic conditions and with the sorbitol containing hypertonic solution suggest that the high concentration of Ca^2+^ is not essential as a stimulus for the recording of LCSs associated with the nucleus. However, we mainly studied LCSs with a high Ca^2+^ concentration in the extracellular solution and this is not a physiological condition. Interpretations and conclusions of results obtained in this condition have therefore limitations when transferred to more physiological states of the fiber. It may be noted that under a hypotonic condition the frequency of cellular LCSs is markedly lower compared to the hypertonic condition with a high Ca^2+^ concentration (Teichmann et al., 2008; Pickering et al., 2009). For future studies, it will be interesting to search for nuclear Ca^2+^ signals using a hypotonic condition.

The results obtained with z-stacks of the nucleus show that at least part of the NLCS is indeed located inside the nuclei although the interpretation of these measurements assumes that small axial shifts due to chromatic errors of the optical system can be neglected. Furthermore, the shape of several NLCSs is restricted by the nuclear borders. These observations suggest that the sources of the NLCSs (at least for part of them) may be located inside the nucleus. It is also possible that Ca^2+^ comes directly from the extracellular space into the nucleus possibly through extensions of the transverse tubular system. In cardiac muscle cells, extensions of the transverse tubular system to the nuclear envelope have been reported (Ibarra et al., 2013). Given the high Ca^2+^ concentration of the hypertonic solution, it is possible that Ca^2+^ comes from the extracellular solution through such extensions of the transverse tubular system. This may lead to the impression that the Ca^2+^ sources are located inside the nucleus. However, such extensions of the transverse tubular system in skeletal muscle fibers could not be observed in a previous study (Jayasinghe and Launikonis, 2013). For a few nuclei we observed membrane structures in the nuclear space (Supplementary Material Image 2). These structures may be a nucleoplasmic reticulum. Further research is needed to demonstrate the existence of a nucleoplasmic reticulum in mammalian skeletal muscle fibers.

Interestingly, several NLCSs and PLCSs were restricted by the borders of the nucleus (Figures 2A,B, 5A and Supplementary Material Image 5; Video 3). There are controversial reports in the literature concerning the permeability of nuclear pore complexes for Ca^2+^ described by Gerasimenko and Gerasimenko (2004). However, nuclear pore complexes are probably Ca^2+^ permeable (Gerasimenko and Gerasimenko, 2004). Nevertheless, there is a report about cytosolic Ca^2+^ signals that are excluded from the nucleus in pancreatic acinar cells (Gerasimenko et al., 1996). In these cells, perinuclear mitochondria isolate nuclear and cytosolic Ca^2+^ signals (Park et al., 2001). Active mitochondria restrict LCSs to the granule region of pancreatic acinar cells (Tinel et al., 1999). In myotubes, mitochondria fine-tune NGCSs (Eisner et al., 2010). In skeletal muscle fibers, mitochondria buffer Ca^2+^ and decrease the frequency of LCSs (Isaeva and Shirokova, 2003). In another study, it has been demonstrated that mitochondria are important for Ca^2+^ signaling after an osmotic shock in mouse skeletal muscle fibers of an animal model of amyotrophic lateral sclerosis (Zhou et al., 2010). We speculate here that perinuclear mitochondria can buffer LCSs also in skeletal muscle fibers and make difficult the diffusion of LCSs from the cytosol to the nucleoplasm and vice versa building a barrier for LCSs between the nucleus and the cytosol. Such a barrier has been described for another cell type by Gerasimenko and Gerasimenko (2004). We explicitly state that there is no experimental data showing that perinuclear mitochondria buffer Ca^2+^ in skeletal muscle fibers and are involved in isolating nuclear and cytosolic LCSs. Some of the NLCSs and PLCSs appear to have a large nuclear component and a small cytosolic component or vice versa (Figure 7B). This may be explained by the limited axial resolution of the optical system and a position near (PLCSs) or in (NLCSs) the nucleus where the nucleus is more arched. On the other hand, these observations could show that part of the LCSs can diffuse to some extent from the nucleoplasm into the cytosol and vice versa. It remains possible that Ca^2+^ diffuses from the cytosol into the nucleus through areas in which there is no barrier for LCSs between the cytosol and nucleus even in the measurements with NLCSs restricted by the borders of the nucleus. Ca^2+^ may then accumulate into the nucleus because of limited extrusion mechanisms in the nucleus even if small amounts of SERCA have been observed in nuclei of polarized MDCK cells (Collado-Hilly et al., 2010). Additionally, our results suggest that there is a difference between PLCSs and NLCSs in the inhibitory effect of dantrolene or nifedipine. PLCSs were stronger inhibited compared to NLCSs in the presence of each blocker. In any case, dantrolene did not decrease the fraction of fibers and nuclei responding with NLCSs. This result suggests that RyRs may be less important for the generation of NLCSs compared to the generation of PLCSs. On the other hand, the activity of NLCSs is low in the presence of dantrolene providing some evidence that RyR may be involved in the genesis of NLCSs. There is no experimental data in the literature showing presence of RyR in the nuclear space of skeletal muscle fibers of adult mammals. The inhibitory effect of the DHPR blocker nifedipine shows that LCSs associated with the nucleus are probably related to the transverse tubular system. In skeletal muscle fibers, the DHPR is important for the activation of the RyR during ec coupling (Felder and Franzini-Armstrong, 2002), but also for the generation of IP_3_ and IP_3_-dependent slow Ca^2+^ signals that occur after ec coupling (Casas et al., 2010); such signals have been related to regulation of gene expression (Jorquera et al., 2013). A DHPR controlled IP_3_ generation may provide an access of DHPR to the regulation of nuclear Ca^2+^ and may be important for the occurrence of NLCSs. The fraction of fibers with NLCSs and PLCSs is not decreased in presence of Xestospongin C. The fraction of nuclei with NLCSs and PLCSs is slightly decreased in the presence of Xestospongin C. These observations suggest that a DHPR controlled IP_3_ generation is not essential for the genesis of NLCSs and PLCSs under the hypertonic conditions used in the present study. In another study, it has been demonstrated that in myotubes a high Ca^2+^ extracellular solution increases the cytosolic and sarcoplasmic Ca^2+^ concentration (Zhou et al., 2006). This effect of the extracellular high Ca^2+^ solution was decreased in the presence of nifedipine (Zhou et al., 2006). A higher cytosolic and sarcoplasmic Ca^2+^ concentration under the hypertonic condition which is reduced in the presence of nifedipine may explain the inhibitory effect of nifedipine in our study. However, the exact mechanism of the DHPR control of NLCSs remains unresolved.

The observed decreased frequency of cellular LCSs in skeletal muscle fibers in the presence of nifedipine is in accordance with a previous study (Pickering et al., 2009). It was recently shown that a crosstalk between the RyR and IP_3_R is required for the generation of LCSs in skeletal muscle fibers of mice after a hypotonic treatment (Tjondrokoesoemo et al., 2013). In our study, the frequency of cellular LCSs was decreased in the presence of Xestospongin C under hypertonic treatment, suggesting again that IP_3_Rs are important for the generation of LCSs but not necessarily for NLCSs or PLCSs. This evidence argues against diffusion of calcium as the cause of the latter. However, we have not excluded a diffusion of Ca^2+^ from the myoplasm into the nucleoplasm. For future studies, it will be interesting to investigate in more detail the possible Ca^2+^ permeability of the nuclear envelope.

Store operated Ca^2+^ entry is another possible mechanism which may be involved in the genesis of LCSs associated with the nucleus in skeletal muscle fibers. This mechanism is not investigated in the present study. Additionally, when using a high Ca^2+^ solution it is possible that there is a leak of Orai1 channels resulting in Ca^2+^ entry from the extracellular space. Orai1 channels are important channels for store operated Ca^2+^ entry (reviewed in Pan et al., 2014). We observed LCSs associated with nuclei in conditions with a Ca^2+^ concentration of 2.5 mmol/l in the extracellular solution. These observations suggest that a leak of Orai1 under hypertonic treatment with a high Ca^2+^ solution is not essential for the genesis of LCSs associated with the nucleus.

The low time resolution of large xyt images used in the present study has limitations for the investigation of fast Ca^2+^ signals such as LCSs. The duration of LCSs cannot be determined precisely. The amplitudes are probably underestimated because of the low frame rate (Steele and Steele, 2014). However, xyt images have also advantages compared to line scanning particularly when LCSs are investigated with regard to cell organelles (Steele and Steele, 2014). In order to explore the temporal properties of PLCSs, we carried out line scans.

The agreement between fibers with NLCSs (when using Fluo-4 AM) identified simultaneously by the algorithm and by the experimenter and only by the experimenter under isotonic and hypertonic conditions (without blocker) is ~73%. The results presented in Table 2 may lead to the impression that the agreement is worse since five fibers with NLCSs identified simultaneously by the algorithm and the experimenter are excluded from the results presented in Table 2 for reasons given in the Results section.

It is possible that satellite cells attached to the skeletal muscle fibers may be activated by the hypertonic condition and the observed Ca^2+^ signals may occur in the satellite cells (when using di-8-ANEPPS for the localization of the nuclei) or in nuclei of the satellite cells (when using di-8-ANEPPS or the DNA dye for the localization of the nuclei) instead of the nuclei of skeletal muscle fibers. Recently, it has been shown that FGF2 increases the Ca^2+^ signals in satellite cells attached to the skeletal muscle fibers (Liu and Schneider, 2014). We recorded NLCSs in nuclei of skeletal muscle fibers of mice, which were not surrounded by a signal of the marker for the satellite cells. Under the used experimental conditions we were able to easily identify satellite cells in the observed preparations. Because of the spectra of the dyes we used the Ca^2+^ indicator Rhod-2 AM for the Ca^2+^ measurements when the marker for the satellite cells was involved. For those reasons Liu and Schneider (2014) used X-rhod-1 AM as a Ca^2+^ indicator in their experiments. Under our experimental conditions, it was more challenging to measure LCSs using Rhod-2 AM compared to Fluo-4 AM. Liu and Schneider (2014) point out that CD34 is present in most quiescent satellite cells and in activated satellite cells (by culture in serum containing media), but that CD34 may not be present in some quiescent satellite cells (Liu and Schneider, 2014). However, we observed NLCSs also in two central nuclei of two skeletal muscle fibers from two mice when using Fluo-4 AM as a Ca^2+^ indicator. Taken together these observations and the results with the satellite cell marker, we conclude that NLCSs exist in skeletal muscle fibers of mice.

We observed also NGCSs in skeletal muscle fibers of mice after the addition of the hypertonic solution. Although it is possible that NGCSs will occur in nuclei of satellite cells, we were not able to measure the NGCSs using the marker for the satellite cells. We observed (when using Fluo-4 AM as a Ca^2+^ indicator) global cellular Ca^2+^ signals in cells in close proximity to skeletal muscle fibers (not attached to skeletal muscle fibers) and they are likely to be satellite cells. On the other hand similar NGCSs were already observed in nuclei of myotubes (Cárdenas et al., 2005) and the NGCS shown in Figure 4C is likely to occur in a nucleus that belongs to a skeletal muscle fiber. It is possible that the NGCSs are spatiotemporal summations of NLCSs. To address this question a better time resolution provided by line scans is required. The NGCSs measured under hypertonic treatment in the absence of a blocker were obtained during the addition of the hypertonic solution which is associated with a shrinkage of the muscle fiber. The shrinkage of the muscle fiber provides a very difficult challenge for records in line scan modus.

There appears to be a heterogeneity between nuclei responding with INCSs. However, we investigated only a fraction of the nuclei of each fiber. For future studies it may be interesting to investigate if there is a different intranuclear Ca^2+^ signaling pattern in nuclei near specific regions of the fiber.

## Conclusions

PLCSs, NGCSs, and NLCSs are present in skeletal muscle fibers of adult mice during a hypertonic treatment. PLCSs occur in a larger fraction of fibers and nuclei compared to NLCSs. NLCSs were found under isotonic conditions as well. These signals may be important in the regulation of adaptive processes as gene expression. The INCSs may be particularly interesting in this respect.

## Funding

This study was supported by the German Ministry for Education and Research (13N7871) (RHAF), Landesforschungsschwerpunkt Baden Württemberg (RHAF), and Chile PIA-ACT1111 (EJ). TG received a Ph.D. scholarship from the Heidelberg Medical School and a scholarship from the German Academic Exchange Service (DAAD Exzellenz II Heidelberg Chile).

## Conflict of interest statement

The authors declare that the research was conducted in the absence of any commercial or financial relationships that could be construed as a potential conflict of interest.

## Abbreviations

LCS(s): localized Ca^2+^ signal(s)
NLCS(s): localized nuclear Ca^2+^ signal(s)
PLCS(s): localized perinuclear Ca^2+^ signal(s)
NGCS(s): global nuclear Ca^2+^ signal(s)
INCS(s): (NLCSs and NGCSs), intranuclear Ca^2+^ signal(s)
CLCS(s): cytosolic localized Ca^2+^ signal(s)
DHPR: dihydropyridine receptor
RyR: ryanodine receptor
IP_3_R: inositol 1,4,5-triphosphate receptor
ec coupling: excitation–contraction coupling
wt mouse: wild type mouse
FWHM: full width at half maximum
FDHM: full duration at half maximum.
