# Supplementary material for: Localized nuclear and perinuclear Ca2+ signals in intact mouse skeletal muscle fibers
Source: Front Physiol. 2015 Sep 29;6:263. doi: 10.3389/fphys.2015.00263 (PMC4586431; doi:10.3389/fphys.2015.00263)
Supplement: Supplementary file 4 [file DataSheet1.DOCX]

***Supplementary Material***

**Localized nuclear and perinuclear Ca^2+^ signals in intact mouse skeletal muscle fibers**

**Tihomir Georgiev*, Mikhail Svirin, Enrique Jaimovich, Rainer H.A. Fink**

*** Correspondence:** Tihomir Georgiev: tihomir.georgiev@physiologie.uni-heidelberg.de

1. **Supplementary Data**

**Measurement procedure**

For the identification of satellite cells, the signal of the satellite cell marker was measured with 488 nm excitation at a spectral range of 500 nm-530 nm. Simultaneously the DNA dye was excited with the 633 nm laser line and the spectral range between 700 nm-800 nm was detected. Under these experimental conditions, the satellite cells could easily be observed and Ca^2+^ measurements were carried out at 543 nm excitation of Rhod-2 AM as Ca^2+^ indicator. To avoid crosstalk, the 488 nm excitation was turned off during the Rhod-2 excitation. The spectral range 550 nm-620 nm or 555 nm-615 nm was detected for the Ca^2+^ signal. At the same time the laser line 633 nm was used for the excitation of the DNA dye and the spectral range 700 nm-800 nm was detected. The structural images are again an average of 8 images.

**Criteria for the determination of the frequencies of cellular LCSs**

For the determination of the relative frequencies of cellular LCSs (fig. 6), the LCSs were identified by the algorithm and may include a few “false positives” in the statistics. This might lead to somewhat higher values of the frequencies in all three cases.

**Image sequences**

**Note:** We provide two different files of each image sequence. The first is a compressed AVI file (video 1-3). The second is a TIFF file (images 3-5) that can be saved and then opened with ImageJ or Fiji.

The image sequences were recorded after the addition of the hypertonic solution.

**Image sequence 1**

LCSs in a compartment (yellow arrow) which is likely a nucleus in a skeletal muscle fiber of a wt mouse. The signal of the Ca^2+^ indicator is green and the signal of the membrane dye is red. The image sequence contains 20 images and the time difference between the images is 0.82 s. Scale bar 10 µm

**Image sequence 2**

Skeletal muscle fiber of a wt mouse stained with a Ca^2+^ indicator (green) and a DNA dye (red). On the left side the Ca^2+^ signal is shown and the yellow marked region is one of the nuclei that can be observed on the merged image (right side). The arrow shows at one of the images with a LNCS. The image sequence contains 11 images and the time difference between the images is 0.82 s. Scale bar 10 µm

**Image sequence 3**

Skeletal muscle fiber of a wt mouse stained with a Ca^2+^ indicator (green) and a DNA dye (red). The arrow shows at a nucleus with PLCSs in the following images. The image sequence contains 16 images and the time difference between the images is 0.82 s. Scale bar 10 µm

1. **Supplementary Image 1**

Transverse tubular system (green) and nuclei (red) in part of a skeletal muscle fiber of a wt mouse. The membrane dye di-8-ANEPPS and the DNA dye HCS NuclearMask Deep Red stain were used. Scale bar 10 µm.

The skeletal muscle fiber was stained with di-8-ANEPPS (40 µmol/l) and HCS NuclearMask Deep Red stain (volume ratio 1/3000) for 25-30 min at 37 °C. After the staining the skeletal muscle fiber was washed with the isotonic solution.

First a 488 nm laser line was used for excitation and the spectral range 500 nm-600 nm was detected. Then the DNA dye was excited at 633 nm and the spectral range 650 nm-800 nm was detected. At the end again the 488 nm laser line was used for excitation and the spectral range 500 nm-600 nm was recorded. No movement of the fiber could be observed. The images are an average of 8 images

1. **Supplementary Image 2**

The measurements in (A), (B) and (C) were recorded under hypertonic treatment.

(A) Part of a skeletal muscle fiber stained with di-8-ANEPPS (first image, green) and the DNA dye (second image, red). The last image is a merge of both signals. The arrow points to a structure in the di-8-ANEPPS signal that is inside the nucleus. Scale bar 5 µm. (B) Part of another skeletal muscle fiber (different animal) stained with di-8-ANEPPS and Fluo-4 AM (signal not shown). A structure in the nuclear space can be seen (arrow). Scale bar 5 µm. (C) Part of a skeletal muscle fiber stained with Fluo-4 AM and the DNA dye (signal not shown). The measurement suggests that there are extensions of the nuclear envelope into the nucleus. Scale bar 5 µm.
