# Supplementary figures and images for: Localized nuclear and perinuclear Ca2+ signals in intact mouse skeletal muscle fibers
Source: Front Physiol. 2015 Sep 29;6:263. doi: 10.3389/fphys.2015.00263 (PMC4586431; doi:10.3389/fphys.2015.00263)

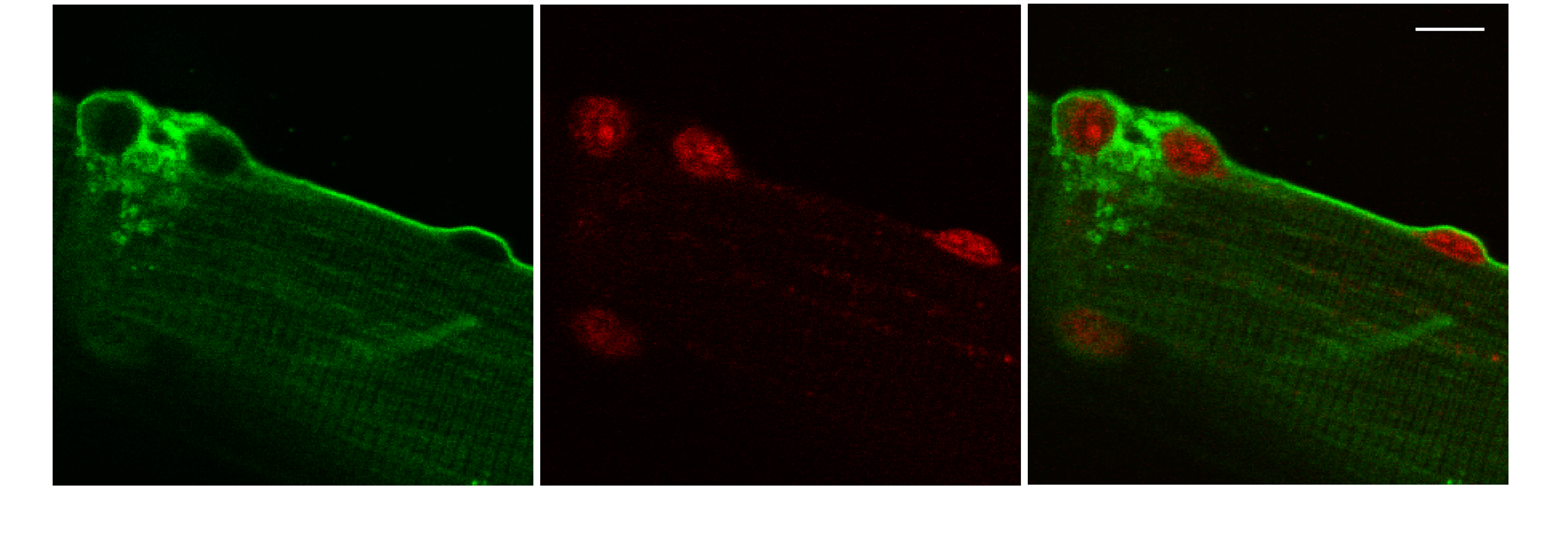

Supplement: Supplementary file 5 [file Image1.TIF]

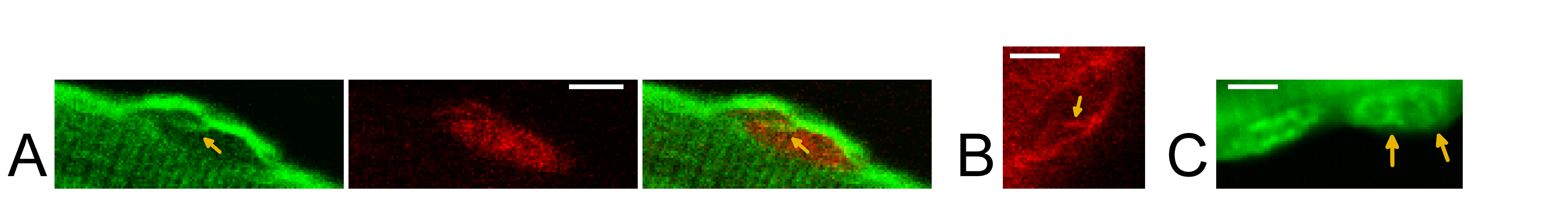

Supplement: Supplementary file 6 [file Image2.TIF]

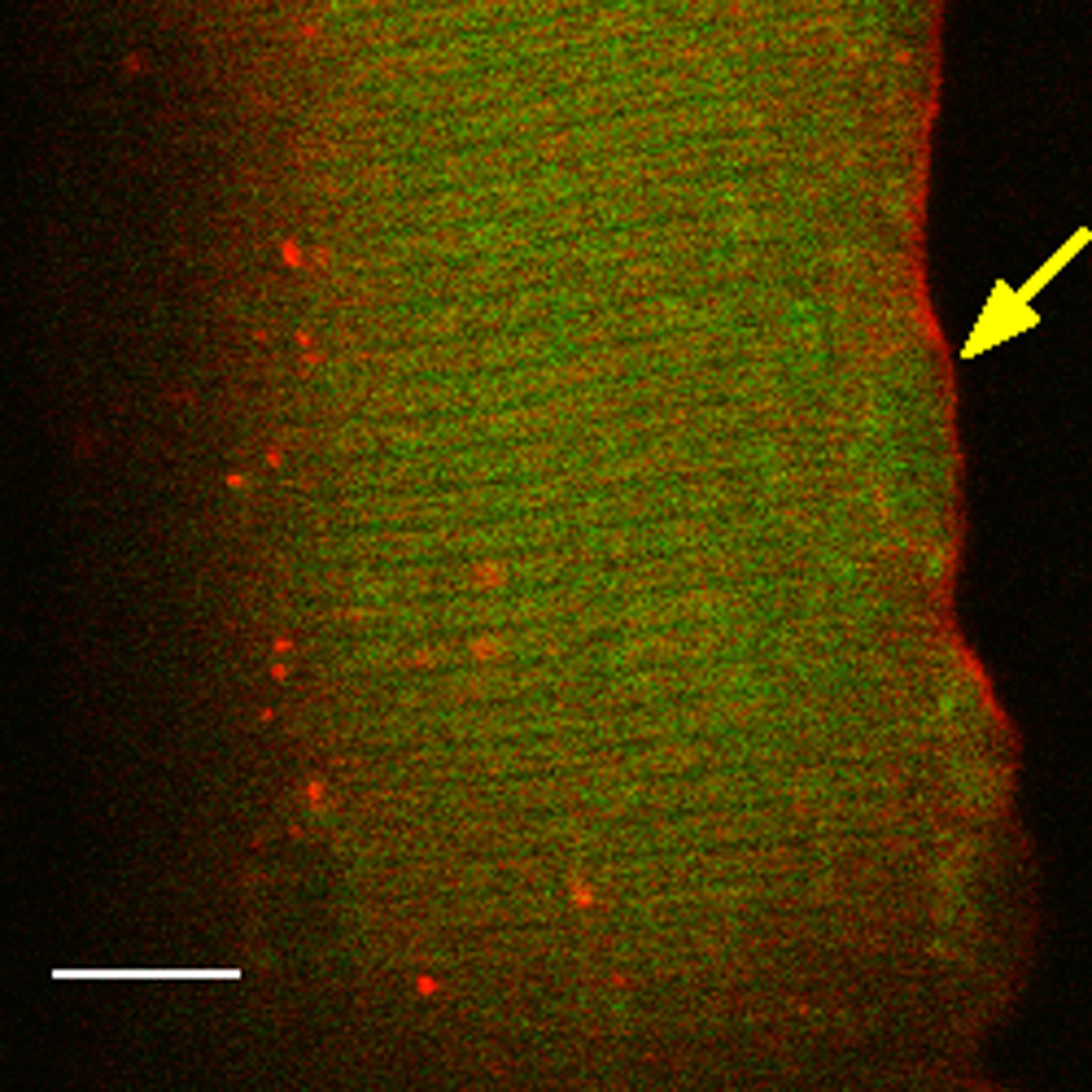

Supplement: Supplementary file 7 [file Image3.TIF]

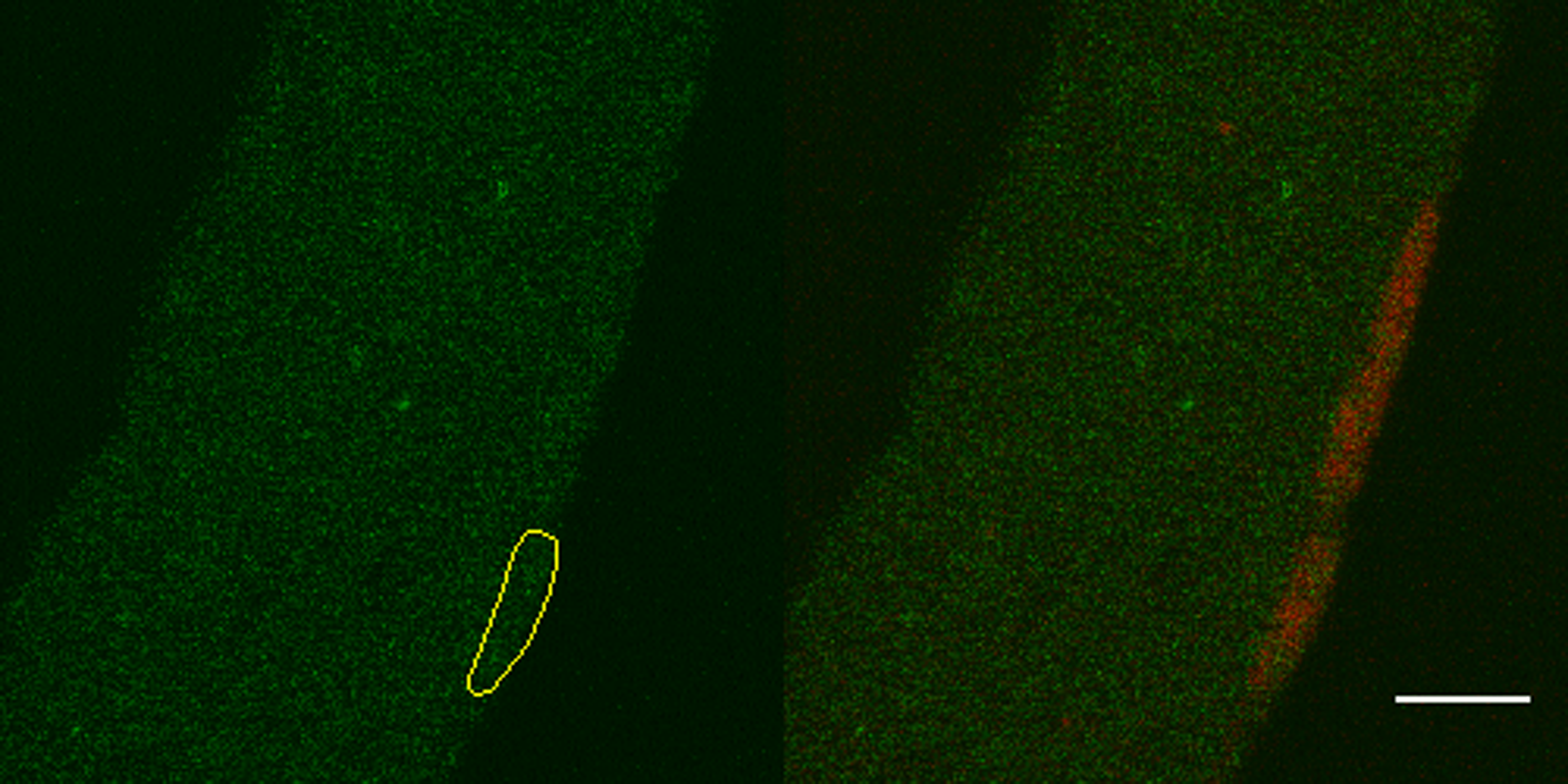

Supplement: Supplementary file 8 [file Image4.TIF]

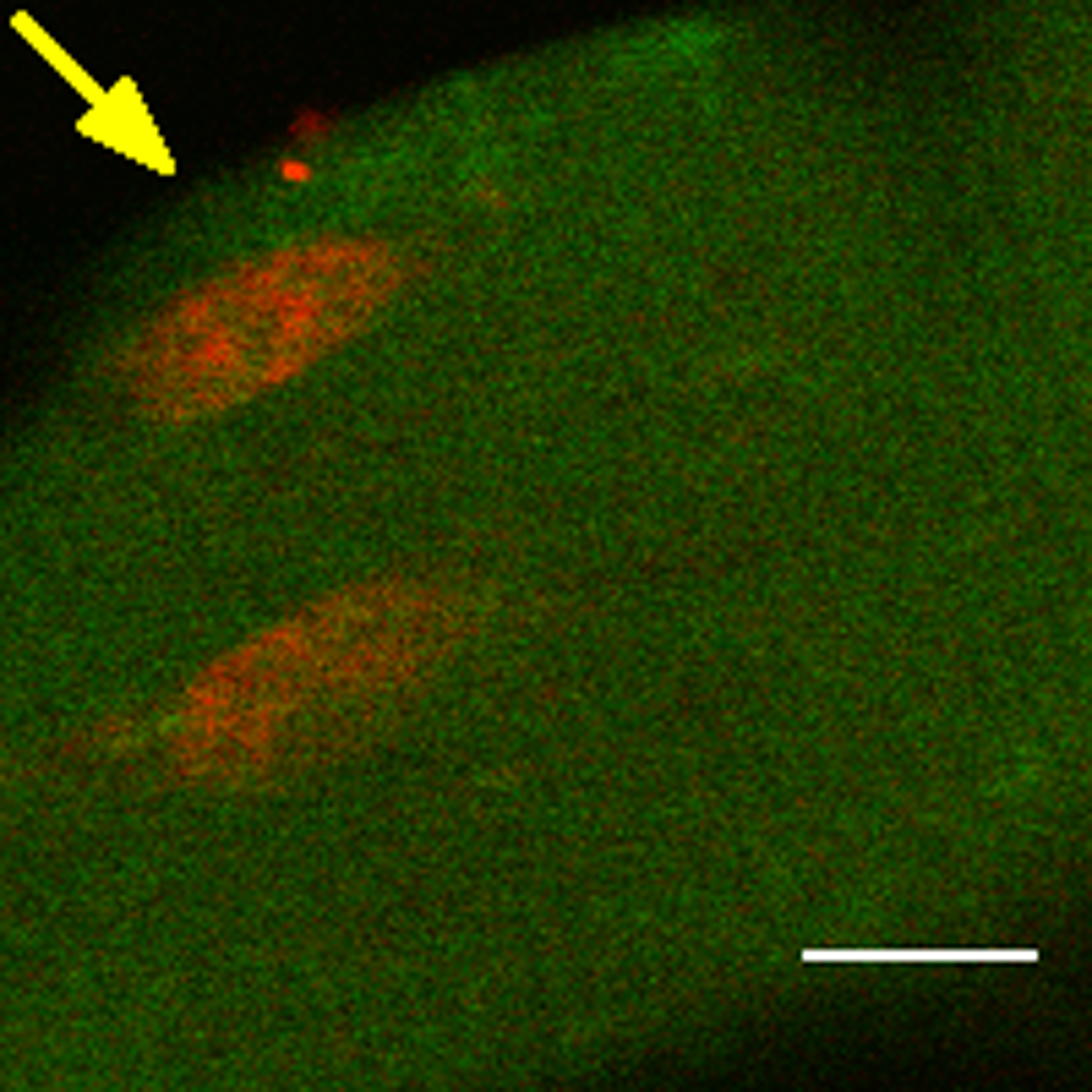

Supplement: Supplementary file 9 [file Image5.TIF]
